# Characterization of EssB, a protein required for secretion of ESAT-6 like proteins in *Staphylococcus aureus *

**DOI:** 10.1186/1471-2180-12-219

**Published:** 2012-09-25

**Authors:** Yi-Hsing Chen, Mark Anderson, Antoni PA Hendrickx, Dominique Missiakas

**Affiliations:** 1 Department of Microbiology, University of Chicago, Chicago, 60637, IL, USA; 2 Department of Microbiology, University of Chicago, 920 E. 58th St, Chicago, IL, 60637, USA

**Keywords:** ESAT-6 secretion, ESS, WXG100, EssB, Type 7 secretion, Staphylococcus aureus

## Abstract

**Background:**

* Staphylococcus aureus * secretes EsxA and EsxB, two small polypeptides of the WXG100 family of proteins. Genetic analyses have shown that production and secretion of EsxA and EsxB require an intact ESAT-6 Secretion System (ESS), a cluster of genes that is conserved in many Firmicutes and encompasses * esxA * and * esxB *. Here, we characterize EssB, one of the proteins encoded by the ESS cluster. EssB is highly conserved in Gram-positive bacteria and belongs to the Cluster of Orthologous Groups of protein COG4499 with no known function.

**Results:**

By generating an internal deletion in * essB *, we demonstrate that EssB is required for secretion of EsxA. We use a polyclonal antibody to identify EssB and show that the protein fractionates with the plasma membrane of * S. aureus *. Yet, when produced in * Escherichia coli, * EssB remains mostly soluble and the purified protein assembles into a highly organized oligomer that can be visualized by electron microscopy. Production of truncated EssB variants in wild-type * S. aureus * confers a dominant negative phenotype on EsxA secretion.

**Conclusions:**

The data presented here support the notion that EssB may oligomerize and interact with other membrane components to form the WXG100-specific translocon in * S. aureus *.

## Background

**Figure 1:**
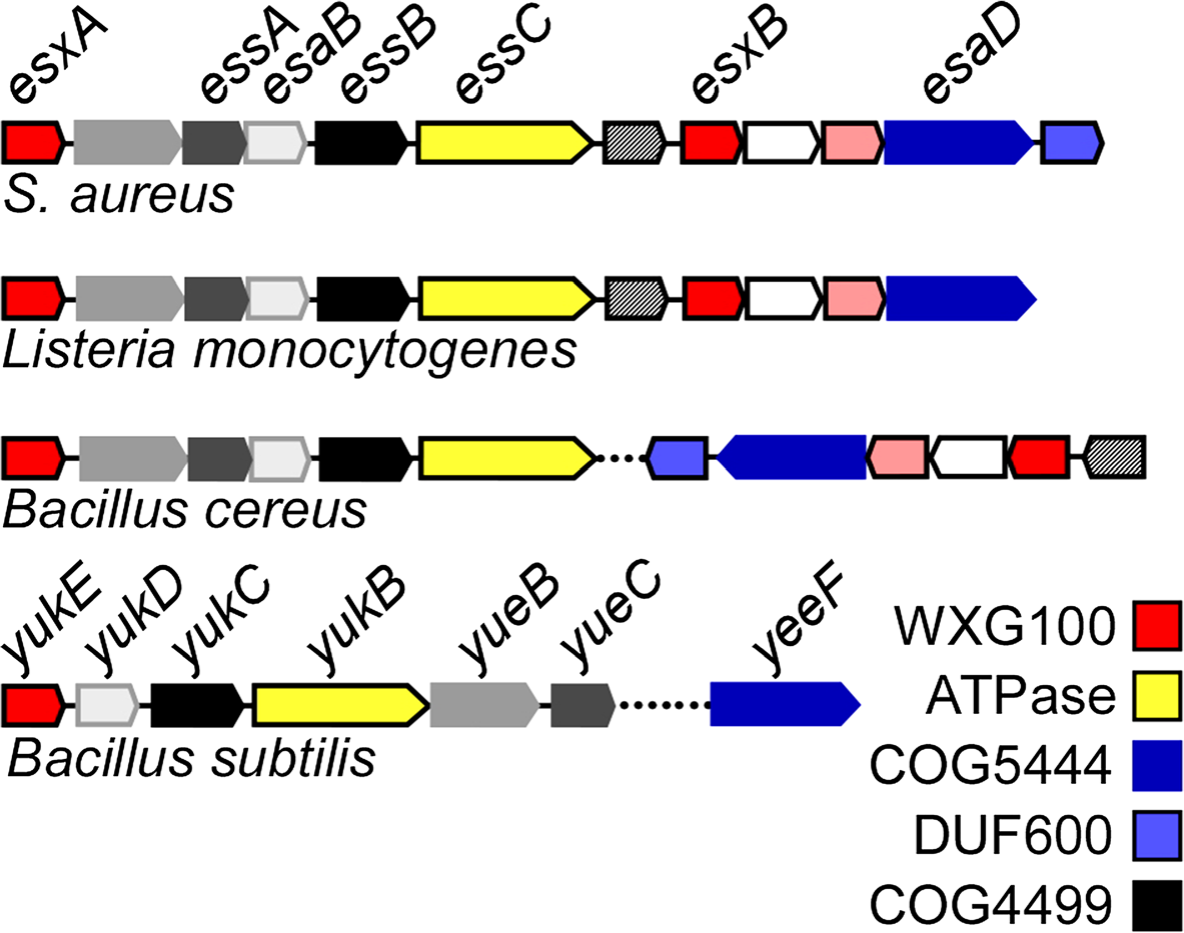
**Schematic of ESS gene clusters in Gram-positive bacteria.** Comparison of the * S. aureus * ESS locus with * Listeria monocytogenes * (strain EGD-e *), Bacillus cereus “cytotoxicus” * (strain NVH391-98) and * B. subtilis * (subsp. * subtilis * strain 168)* . * Genes sharing sequence homology are depicted with the same color. Proteins with defined conserved domains are indicated as follows: WXG100 family of proteins (red), FtsK SpoIIIE-like ATPases (yellow), Cluster of Orthologous Groups of proteins COG5444 (dark blue), COG4499 (black) and proteins with a Domain of Unknown Function DUF600 (light blue). Dashed lines between blocks of genes indicate that the genes are not found in close proximity but elsewhere on the same chromosome. The nomenclature for the * S. aureus * cluster has been described [20]. The genetic organization is conserved in * S. aureus * strains. Gene names for * B. subtilis (subsp. Subtilis strain 168) * are annotated as described in the National Center for Biotechnology Information databank.

In Gram-positive bacteria, proteins released in the extracellular environment are synthesized as precursor polypeptides with a cleavable N-terminal leader peptide as the sole topogenic signal. Precursors are moved across the plasma membrane by a translocon and signal peptidases act on newly translocated precursors to release the mature polypeptide from the membrane [1]. The events leading to protein translocation across the plasma membrane have been genetically dissected using the model organism * Escherichia coli *. Most precursor proteins travel in an unfolded state through the SecYEG translocon [2-5], pushed by the cytoplasmic ATPase SecA [6]. Precursor proteins bearing a leader peptide with the twin-arginine motif are moved across the plasma membrane by the Tat translocon [7,8]. Recently, it has been observed that some bacteria, in particular Firmicutes and Actinobacteria, can secrete proteins lacking a canonical leader peptide [9]. Many of these proteins share some distinguishing and conserved features that include small size (approximately 100-amino acid residues), a WXG amino acid motif in the middle of the protein [10] and a conserved three-dimensional structure (helix–turn–helix hairpin) [11,12]. Together, these proteins form the WXG100 family of proteins [10]. ESAT-6 and CFP-10 of * Mycobacterium tuberculosis * are the founding members of the WXG100 family of proteins and are identified with the acronym EsxA and EsxB for ESAT-6 extracellular protein A and B[10]. Bioinformatic and genetic approaches have revealed that the * esxA * and * esxB * genes cluster with both conserved and non-conserved genes of unknown function that are required for the stability and secretion of WXG100/Esx proteins into the extracellular milieu [13-16]. These clusters are conserved among several Firmicutes (Figure 1) but not with Mycobacteriaceae who only share EssC-like ATPases [10,17]. The name ESX has been used to refer to such gene clusters in Mycobacteriaceae and * M. tuberculosis * for example encodes five ESX clusters (ESX-1 through ESX-5) [17]. In more general term, ESX mediated secretion has been refereed as Type 7 secretion but it was noted that this general designation should not be used for Firmicutes owing to the lack of overall sequence conservation [18]. Clusters bearing * esx * genes have therefore been referred as ESAT-6 Secretion Systems (ESS) in * Staphylococcus aureus * and * Bacillus anthracis * where they have been experimentally examined [16,19-21] and sometimes as WXG100 Secretion Systems (WSS) [22]. It has been proposed that at least three factors, ESAT-6 secretion system genes A, B and C (EssA, EssB and EssC), are important for secretion of WXG100 proteins in * S. aureus * based on the phenotype of transposon insertions in the three corresponding genes [16]. Here, we present genetic and biochemical data that support this hypothesis for EssB. By generating a minimal deletion of * essB * in strain USA300* , * we observe that EsxA remains in the cytoplasm and is no longer secreted into the extracellular milieu. Further, we demonstrate that EssB localizes to the plasma membrane of * S. aureus * and that truncated variants of EssB confer a dominant-negative phenotype on chromosomally encoded EssB (loss of EsxA secretion). These results are consistent with the notion that EssB oligomerizes and/or interacts with a larger complex of proteins to mediate translocation of EsxA across the plasma membrane of * S. aureus *. 

## Results

### EssB is required for the secretion of EsxA by * S. aureus * USA300

**Figure 2:**
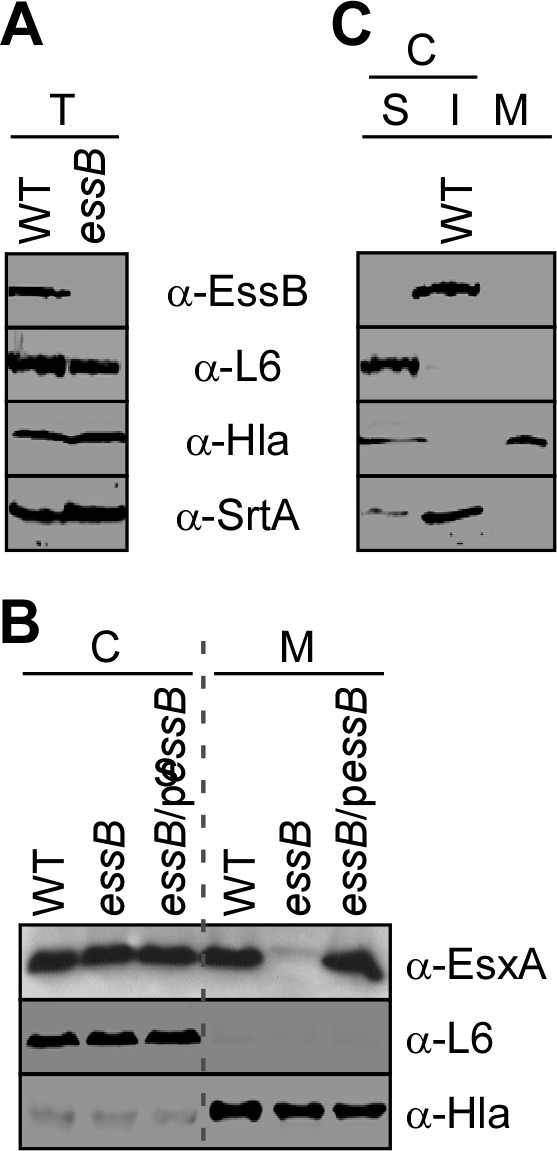
**Identification and characterization of EssB.** (**A**) * S. aureus * USA300 (WT) or isogenic mutant * essB * were examined for production (T: total culture extracts) and subcellular localization of EssB (C: cell extracts followed by 100,000 x * g * sedimentation and separation of soluble, S and insoluble I proteins; M: medium). Proteins in each fraction were precipitated with trichloroacetic acid, separated by SDS-PAGE and detected by immunoblotting with specific antibodies [α-EssB, as well as α-L6, α-Hla, α-SrtA, as cytoplasmic, secreted and membrane protein controls, respectively]. (**B**) Plasmid complementation analysis of bacterial cultures separated between cells (C) and medium (M). * S. aureus * USA300 (WT) or * essB * mutants harboring or not a complementing plasmid (p* essB *) were examined for their ability to secrete EsxA in the culture medium. Samples were analyzed as in panel A.

The ESS pathway has previously been examined in * S. aureus * strain Newman, where a transposon insertion in gene NWMN_0222 resulted in a severe loss of EsxA and EsxB production. A definitive function for the * ess * gene product in * S. aureus * Newman could not be revealed, owing to the instability of EsxA and EsxB in this strain. Nevertheless, it was hypothesized that NWMN_0222 may contribute to the secretion of EsxA and EsxB across the membrane. The gene was named EssB for ESAT-6 like secretion system gene B. Further examinations revealed low expression of the ESS cluster in * S. aureus * Newman as compared to the more virulent staphylococcal isolates * S. aureus * USA200, USA300 and USA400 [19,20]. We therefore sought to study the secretion of EsxA in strain USA300 and generated an * essB * mutant via allelic replacement. This mutant harbors an internal deletion by fusing the first fifteen and last fifteen codons of the * essB * open reading frame, which otherwise encodes a 444 amino acid polypeptide. In parallel, we produced recombinant EssB in * E. coli * (see below) and used the purified protein to generate a polyclonal rabbit serum. Cultures of wild-type * S. aureus * USA300 and the isogenic * essB * mutant were grown to mid-log phase and treated with lysostaphin to generate total protein extracts (T, as shown on Figure 2A). Proteins were precipitated with trichloroacetic acid and separated on SDS/PAGE followed by transfer to PVDF membrane for immunoblotting. Blots shown on Figure 2A identify an EssB-immune reactive species in * S. aureus * USA300 that is absent in the extract of the * essB * mutant. As a control, ribosomal protein (L6), α-hemolysin (Hla) and sortase A (SrtA) were identified in all extracts. The EssB immune species migrated at about 52 kDa on SDS/PAGE. To evaluate the phenotype of the * essB * mutant, staphylococcal cultures were centrifuged to separate bacterial cells (C) from the medium (M), and proteins in both fractions were examined by immunoblotting with EsxA-specific rabbit antibodies (Figure 2B). EsxA was found in bacterial cells and in the extracellular medium of * S. aureus * USA300 cultures. In contrast, EsxA remained in the cytoplasm of * essB * mutant staphylococci (Figure 2B)* . * EsxA immune reactive signals were reduced to non-detectable levels in the extracellular milieu of an * essB * mutant, supporting the notion that EssB is required for the secretion of EsxA. The deletion of the * essB * gene did not affect the localization of the ribosomal protein L6 in the cytoplasm or the secretion of Hla into the extracellular medium (Figure 2B). EsxA secretion was restored to wild-type levels when * essB * was expressed from a plasmid (p* essB *), suggesting that deletion of the * essB * gene does not affect the expression of downstream genes also involved in the ESS pathway [16,19,20]. 

### Subcellular localization of EssB

We wondered whether EssB is itself secreted or localizes to a particular subcellular compartment (cytosol/membrane). A culture of * S. aureus * USA300 was centrifuged to separate cells from the extracellular milieu. As expected Hla, but not EssB, was found in the extracellular medium (Figure 2C; lane M). Further fractionation was achieved by subjecting lysed cellular extracts to sedimentation at 100,000 × * g *. As a control for subcellular fractionation, samples were examined by immunoblot for the ribosomal protein L6 (S, soluble) and membrane protein SrtA (I, insoluble). EssB was identified in the membrane sediment along with SrtA (Figure 2C), suggesting that EssB may either be inserted into the lipid bilayer or associated with one or more proteins in the membrane. This finding is in good agreement with a recent report suggesting that YukC the * B. * subtilis homologue of EssB (Figure 1) belongs to the membrane proteome of * B. subtilis *[23].

The TMHMM algorithm (http://www.cbs.dtu.dk/services/TMHMM-2.0) was used to perform sequence-based prediction of EssB, which identified a string of hydrophobic residues amino acids 229–251 (W^229^VAIGMTTLSVLLIAFLAFLYFS^251^) at the center of the EssB polypeptide. Hereafter we refer to the segment of hydrophobic amino acids within EssB as the Putative Trans Membrane Domain (PTMD).

### Deleting * essB * affects the production of several ESS factors

**Figure 3:**
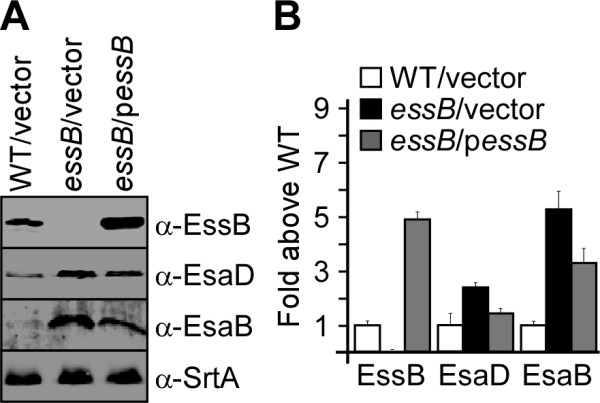
**Loss of EssB affects production of EsaB and EsaD.** (**A**) Total culture lysates of WT (USA300) and * essB * mutant with the empty vector control (vector) or complementing plasmid (p* essB *) were examined for the production of EssB, EsaB and EsaD using polyclonal antibodies and as described in the legend of Figure 2A. The experiment was repeated three times in duplicate and bands corresponding to immune reactive species were scanned and quantified using a Li-Cor Biosystems Odyssey imager. Quantification of the data is shown in panel **B**.

Recently, we reported that the last gene of the ESS cluster, * esaD, * is required for the effective secretion of EsxA (Figure 1) [20]. We therefore wondered whether the EsxA secretion phenotype of the * essB * mutant could be explained by the possible loss of expression of other EsaD factors. To examine this possibility, extracts of bacterial cultures (medium and lysed cells) derived from wild-type or the * essB * mutant carrying either a plasmid control without insert (vector) or the complementing plasmid (p* essB *), were subjected to immunoblot analysis using antibodies against EsaD as well as the control protein SrtA (Figure 3A). Interestingly, EsaD appeared to accumulate in the * essB * mutant. Intrigued by this finding, we performed a similar analysis using antibodies against EsaB, a small cytoplasmic protein that modulates the ESS pathway by an unknown mechanism [19]. EsaB is conserved in the minimal ESS cluster of * B. subtilis * where it is designated YukD (Figure 1). We observed that deletion of * essB * also led to the accumulation of EsaB (Figure 3A). These observations were quantified by performing each experiment in triplicate and comparing the average abundance of proteins in wild-type and * essB * mutant strains. EsaD and EsaB were found to accumulate with 2.5-fold and 5-fold increase over wild type, respectively (Figure 3B). Expression of wild-type * essB * from the complementing plasmid rescued this phenotype, albeit that only partial complementation was achieved. Perhaps, the physiological ratio between EssB and EsaB could not be achieved upon overexpression of * essB * using a plasmid. Taken together, these observations suggest that EssB is a critical component of the ESS pathway required for secretion of EsxA and proper accumulation of EsaB and EsaD. 

### Recombinant EssB is soluble and prone to multimerization

**Figure 4:**
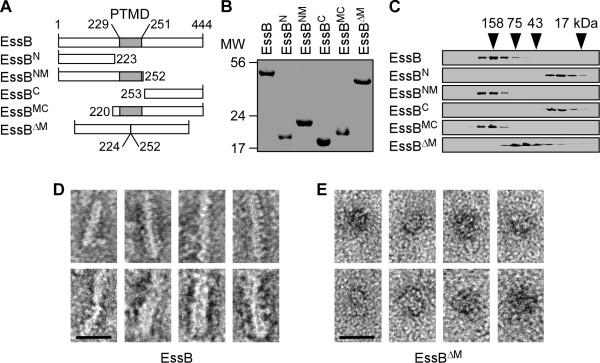
**Purification and characterization of recombinant EssB and truncated variants.** (**A**) Diagrammatic representation of full length EssB and truncated variants produced in * E. coli. * Numbers indicate amino acid positions in the primary sequence and the grey box labeled PTMD depicts the hydrophobic sequence. (**B**) Coomassie gel of purified recombinant proteins as shown in panel A. Proteins were purified from * E. coli * by affinity chromatography and affinity tags were removed. (**C**) Size exclusion chromatography of full length EssB and truncated variants shown in panel B. Proteins (~100 μg) were loaded onto a Superdex^TM^ 75 10/300 GL and fractions (0.5 ml) were collected and analyzed by SDS-PAGE. Proteins in the gel were visualized by Coomassie staining. Masses of protein standards used for calibration are shown above the gels (158, 75, 43, 17 kDa) and correspond to the exclusion volumes of Aldolase, Conalbumin, Ovalbumin and Myoglobin, respectively. (**D-E**) TEM of purified recombinant EssB (**D**) and EssB^ΔM^ (**E**). The proteins were allowed to bind to glow discharged grids and were negatively stained using 2% uranyl acetate. This analysis reveals a rod-like structure for EssB and more spherical, aggregated-like structure for EssB^ΔM^. Scale bar = 20 nm.

EssB is a 444 amino acid protein with relative molar mass * M *_r_ 52023.94 (Figure 4A)*.* Its production could be achieved to high yield in * E. coli * BL21(DE3) harboring pET15b encoding * essB. * In order to purify the protein, cells were lysed in a French pressure cell and lysates were subjected to ultracentrifugation at 100,000 × * g * for 60 min. To our surprise most EssB remained in the supernatant (>75%). Assuming that amino acids 229–251 represent a hydrophobic buried segment, the primary sequence of EssB can be roughly divided in two soluble N-terminal and C-terminal domains (Figure 4A). We generated five recombinant variants encompassing the predicted soluble N- or C-terminal domain with or without the PTMD as well as a variant lacking PTMD (Figure 4A). The variants were named EssB^N^, EssB^C^, EssB^NM^, EssB^MC^, EssB^ΔM^, respectively. Similar to full length EssB, over 75% of the overproduced proteins could be recovered from the supernatant of * E. coli * lysates subjected to ultracentrifugation (100,000 × * g * for 60 min) with the exception of EssB^ΔM^ that was poorly expressed. Full length EssB along with all variants were purified to homogeneity using affinity chromatography and the affinity tags were removed by thrombin digestion. The purity of the polypeptides was evaluated on Coomassie-stained SDS/PAGE (Figure 4B). Next, these polypeptides were subjected to gel filtration onto Sephacryl S-200 column and aliquots of eluted fractions were evaluated once more on Coomassie-stained SDS/PAGE (Figure 4C). When subjected to gel filtration, EssB eluted as a homogenous peak with * M *_r_ ~ 158,000 (Figure 4C). The elution profile did not change when the protein concentration was increased or decreased by a factor of 10 and EssB protein did not scatter UV light suggesting that the polypeptide remained soluble (not shown). Variants that lacked PTMD, EssB^N^ and EssB^C^, eluted with * M *_r_ of ~22-25,000, close to their calculated masses (Figure 4C). In contrast, variants that retained PTMD, EssB^NM^ and EssB^MC^, eluted with * M *_r_ >158,000 following size exclusion chromatography (a somewhat higher mass than the full length protein). Removal of PTMD caused EssB^ΔM^ to elute with a * M *_r_ of ~47,000 suggesting that quite like EssB^N^ and EssB^C^, this variant did not multimerize (Figure 4C).

Visualization of purified EssB protein by transmission electron microscopy suggested that the sample is homogenous. Small dense structures could be seen throughout the field and at larger magnification they revealed a clear rod-shaped organization of the molecule (Figure 4D). A similar analysis was performed for affinity purified EssB^ΔM^. Transmission electron micrography revealed that overall the protein preparation was homogeneous (not shown), however the rod-shaped structure of EssB is lost in this variant (Figure 4E). Together, these results suggest that the PTMD segment is required for the multimerization of EssB and that the rod-shaped structure may be an energetically favorable conformation in the cytoplasm of * E. coli *. Interestingly, the structure for a so-called “cytoplasmic component of EssB” has been deposited in the databank and made publicly available (http://www.ncbi.nlm.nih.gov/Structure/mmdb/mmdbsrv.cgi?Dopt=s&uid=99898, http://www.rcsb.org/pdb/explore/explore.do?pdbId=4ANN). This component encompasses the first 215 amino acids of EssB and behaves as a soluble monomer quite like EssB^N^ examined in this study.

### Truncated EssB variants display a dominant negative phenotype in * S. aureus *

**Figure 5:**
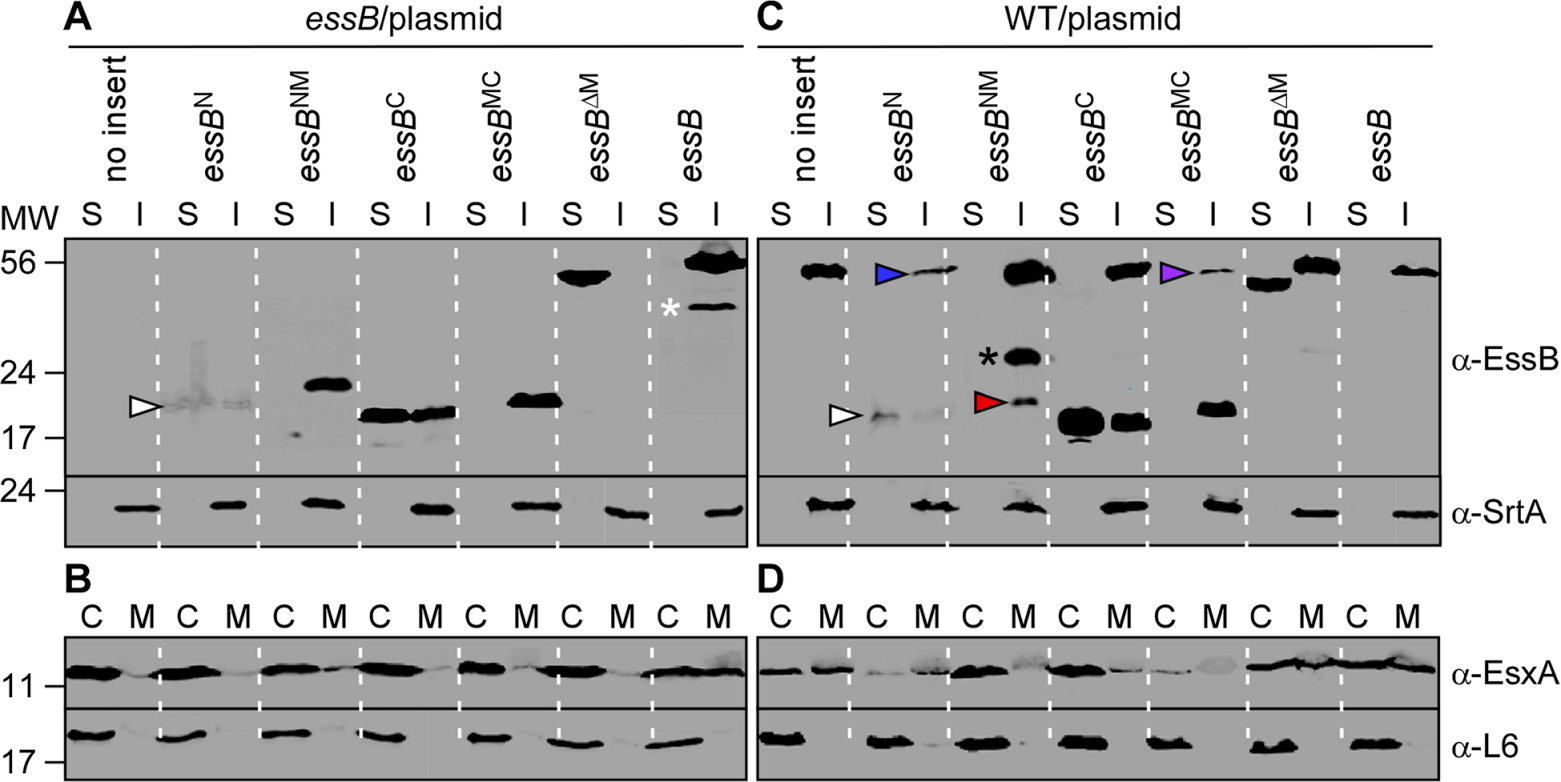
**Complementation and dominant negative activity of truncated EssB variants.** (**A-B**) Complementation studies. * S. aureus * USA300 lacking functional * essB * was transformed with vector carrying either no insert, or various truncated variants of EssB or full length EssB. (**A**) The subcellular localization of EssB immune reactive species was assessed by subjecting cell lysates to ultracentrifugation to separate soluble (S) and (I) insoluble proteins and proteins in both extracts were resolved by SDS-PAGE followed by immunoblotting with specific antibodies (α-SrtA is used for subcelluar fractionation control of an insoluble membrane protein). (**B**) Cultures were examined for production and secretion of EsxA. Cultures were spun to separate proteins in cells (C) from secreted protein in the medium (M). α-L6 is used for fractionation control of a cytosolic protein. (**C-D**) Dominant negative studies. Truncated variants of EssB were examined for protein localization (**C**) and EsxA secretion (**D**) as described in panel **A**. All plasmids were transformed in wild-type strain USA300 (WT). All truncated variants with the exception of EssB^ΔM^ lacking PTMD prevented secretion of EsxA. The data for a duplicate of three independent experiments are shown. Arrows indicate proteins with correct mass found in reduced abundance (white arrow: EssB^N^; red arrow: EssB^NM^; blue and purple arrows: endogenous EssB). Protein products with aberrant mass are depicted with asterisks.

We wondered whether truncated EssB variants may trigger misassembly of the ESS secretion machinery and interfere with the secretion of EsxA in * S. aureus *. To test this, the EssB variants illustrated in Figure 4A were cloned into the expression plasmid pWWW412 and transformed into * S. aureus * USA300 wild-type and * essB * mutant strains. First, complementation of Ess function was assessed in the * essB * mutant, using plasmids carrying either no insert or wild-type * essB * controls or * essB * variants encoding EssB^N^, EssB^C^, EssB^NM^, EssB^MC^, EssB^ΔM^, respectively (Figure 5A). Cell extracts were fractionated to reveal synthesis and subcellular localization of full length or truncated EssB proteins following sedimentation of lysed cells at 100,000 × * g * (Figure 5A). As a control, sortase A (SrtA) was found in the sediment (I, insoluble fraction) of ultracentrifugation samples. EssB protein expressed from p* essB * in the * essB * mutant strain also sedimented during ultracentrifugation (Figure 5A; right two lanes) similar to endogenous EssB from * S. aureus * USA300 (Figure 2A). An additional immune reactive species was observed when EssB was overproduced from the plasmid (Figure 5A, white asterisk). Variants carrying the PTMD sequence, EssB^NM^ and EssB^MC^, sedimented during ultracentrifugation, whereas EssB^ΔM^, the variant that lacks the PTMD sequence, did not. Two proteins assumed aberrant behavior. The EssB^N^ protein was either poorly produced or very unstable in * S. aureus essB * mutant (Figure 5A; white arrow). EssB^C^ partitioned into both the soluble and the insoluble fractions. Perhaps, this domain interacts weakly with components of the secretion machine embedded in the membrane. Of note, only the plasmid encoding full-length EssB restored EsxA secretion into the extracellular medium of * essB * mutant cultures (M); all other plasmids failed to complement * essB * for EsxA secretion (Figure 5B). As expected, the control ribosomal protein L6 was found in cell lysates (C) (Figure 5B).

When transformed into wild-type * S. aureus * USA300, plasmid produced EssB and variants fractioned as before following 100,000 × * g * ultracentrifugation (Figure 5C). Briefly, EssB, EssB^NM^ and EssB^MC^ were found in the sediment, EssB^ΔM^ remained soluble and EssB^C^ fractionated equally in the soluble and insoluble compartments (Figure 5C). Expression of EssB^NM^ led to some degradation of EssB (Figure 5C, black asterisk). As before, very little EssB^N^ immunoreactive material could be detected in * S. aureus * USA300 cells (Figure 5C, white arrow). Interestingly, its production caused a reduction of wild-type EssB (Figure 5C, blue arrow). EssB was also unstable in the merodiploid strain expressing EssB^MC^ (Figure 5C; purple arrow). Not surprisingly, destabilization of EssB by either EssB^N^ or EssB^MC^ led to altered expression and secretion of EsxA (Figure 5D). Sedimentable variants encompassing the PTMD, EssB^NM^ and EssB^MC^, caused a dominant-negative phenotype on the activity of wild-type EssB and as a result expression or secretion of EsxA were altered. On the contrary, EssB^ΔM^ lacking PTMD remained soluble and did not interfere with EssB function. Taken together, these data suggest that EssB variants that sediment with staphylococcal membranes interfere with the stability or function of endogenous EssB and as a consequence EsxA production and secretion are also affected. Thus, EssB is part of the secretion machine and its multimerization and possible association with other Ess components enables the secretion of EsxA.

## Discussion

Secreted proteins are generally tagged with topogenic sequences for recognition by a specific secretion machine and transport across the plasma membrane. Over a third of all proteins synthesized by a bacterial cell carry leader peptides, the topogenic signal for recognition by the Sec machine [24]. The corresponding * sec * genes are scattered on the chromosome although their gene products assemble specifically at the membrane to mediate the faithful secretion of a variety of polypeptides. Bacteria have also evolved highly specialized secretion systems for the transport of specific proteins across lipid bilayers and organized the genes encoding machine components and their substrates into clusters whose expression is controlled by adjacent transcriptional units [25,26]. The * S. aureus * ESS cluster represents one such dedicated secretion pathway. ESS genes are encoded within an eleven gene cluster and when deleted impair the production or secretion of small proteins with the WXG amino acid signature. Here, we have begun the characterization of EssB, one of the proteins of the staphylococcal ESS cluster (Figure 1).

Bioinformatic searches revealed that EssB is found in Gram-positive bacteria that harbor ESS gene clusters closely related to the staphylococcal ESS pathway (Figure 1). The protein belongs to the Cluster of Orthologous Groups of protein COG4499 and is annotated as a predicted membrane protein homologous to * B. subtilis * YukC (Figure 1). COG4499 protein members are all arranged in a single architecture meaning that the entire protein defines a single domain that is never truncated nor fused with another protein domain. By performing a Position-Specific Iterative BLAST (PSI-BLAST) in NCBI (under default conditions within two iterations), an obvious homology can be identified between the EssB/YukC family of proteins and the TraF proteins from Gram-positive conjugative plasmids [27]. This is interesting (yet perplexing) because it has been proposed that the specialized secretory apparatus ESX-1 of * M. smegmatis * that lacks an EssB/YukC/TraF homologue carries out DNA transfer [28].

By raising a polyclonal antibody against EssB, we find that the protein sediments with * S. aureus * membranes in a manner similar to SrtA, a well-characterized membrane embedded protein [29]. Residues 229–251 roughly define a hydrophobic sequence reminiscent of a transmembrane spanning segment (PTMD). Interestingly, recombinant EssB behaves as a soluble oligomer in * E. coli * with a rod-shaped like structure and the PTMD sequence appears to be necessary and sufficient for this oligomerization process. Obviously, this conformation may simply represent an energetically favorable state for an otherwise membrane-spanning. Nonetheless, recombinant EssB^NM^ and EssB^MC^ are more prone to multimerization than intact EssB suggesting that the full-length sequence limits or regulates the oligomerization of the protein. Protein translocators of other secretion systems such as the Tat or holin pathways undergo regulated multimerization to facilitate pore function in the membrane [30,31]. In * S.aureus *, the presence of the PTMD targets EssB^NM^ and EssB^MC^ to the membrane. This targeting appears to affect the function of endogenous EssB in wild-type staphylococci. On the contrary, EssB^ΔM^ (lacking PTMD) is soluble. It is unable to complement the * essB * mutant and it displays no dominance over wild-type for EsxA secretion. As such, none of the truncated EssB variant could complement wild-type EssB for secretion. Further studies are needed to determine whether the PTMD sequence serves as an autonomous membrane-spanning domain or whether it provides a mean to associate with another integral membrane protein encoded within the ESS cluster.

Deletion of * essB * in strain USA300 leads to loss of EsxA secretion and EsxA remains in the cell. Because overproduction of EssB is not toxic in * E. coli *, we do not believe that this protein alone is capable of forming a pore for the passage of secreted substrates. Interestingly, two other proteins EsaB and EsaD also accumulate in the * essB * mutant. While the exact role of EsaB is still unknown, it does not appear to be a secreted substrate [19], and thus the reason for this increase is unclear but it points to additional biochemical interactions within proteins of the ESS cluster. Recent evidence suggests that EsaD is a membrane protein also required for EsxA secretion [20]. Perhaps EssB interacts physically with EsaD to either complete or regulate formation of the translocon. Future studies are needed to address this possibility and determine whether EssB is an integral or peripheral element of the ESS translocon.

## Conclusions

The ESS pathway is an alternate and conserved secretion system of several Gram-positive bacteria. Here, we show that EssB is found in the membrane of * S. aureus * and deletion of the corresponding gene abrogates secretion of EsxA. We show that a hydrophobic segment in the middle of the protein referred as PTMD is required for targeting to the plasma membrane. We observe that recombinant EssB harboring PTMD folds into an oligomeric rod-shaped structure that allows the protein to remain soluble in * E. coli. * Interestingly, truncated EssB variants harboring an intact PTMD display a dominant negative phenotype over wild type EssB for secretion of EsxA. The data indicate that EssB is an essential component of the ESS translocon and likely interacts with itself and other machine components. Together, this study provides the first genetic and biochemical characterization of the ESS translocon in * S. aureus *.

## Methods

### Growth conditions

* S. aureus * and * Escherichia coli * cultures were grown at 37° in tryptic soy (TS) with 0.2% serum or Luria Bertani (LB) broth or agar, respectively. Chloramphenicol and ampicillin were used at 10 and 100 μg/l for plasmid selection, respectively.

### Bacterial strains and plasmids

* S. aureus * strain USA300 was obtained through the Network on Antimicrobial Resistance in * S. aureus * (NARSA, NIAID). For deletion of * essB, * a 2-kbp DNA fragment flanking the * essB * gene and carrying the first and last fifteen codons of * essB * gene was amplified by PCR, with abutted * Bgl *II restriction site (See Table 1 for sequences of oligonucleotides used in this study). The DNA fragment was cloned into pKOR1 for allelic replacement performed as described earlier [32]. The * E. coli – S. aureus * shuttle vector pWWW412 that carries the * hprK * promoter and Shine-Dalgarno sequence (275bp upstream of the * hprK lgt yvoF yvcD * translational start site) and three cloning sites * Nde *I, * Xho *I, * BamH *I, as described earlier [33] was used for expression of wild-type * essB * and truncated variants in * S. aureus *. All cloning procedures were carried out in * E. coli * and ampicillin was used at 100 μg/l for plasmid selection. Plasmids were electroporated into * S. aureus * RN4220 prior to introduction into * S. aureus * USA300. The complementation plasmids p* essB * has been described earlier [20]*.* All truncated variants were generated by amplification of DNA sequences using PCR and primer pairs with sequences listed in Table 1. For deletion of the Putative Trans Membrane Domain (PTMD), two DNA fragments were amplified with two sets of primers prior to ligation in pWWW412. The pET15b (Novagen) and pGEX-2T (GE Healthcare) vectors were used for expression of recombinant * essB * and truncated variants in * E. coli *. The DNA sequences of the full-length gene and variants were amplified by PCR using primers listed in Table 1. Vector pET15b was used for production of recombinant EssB, EssB^NM^, EssB^MC^, EssB^ΔM^, and pGEX-2T for production of recombinant EssB^N^ and EssB^C^. All clones were validated by nucleotide sequencing performed by the DNA Sequencing Facility of the Cancer Research Center at the University of Chicago. All plasmids and strains are listed in Table 2. 

**Table 1:** Oligonucleotides used in this study

**Name**	**Nucleotide sequence**	**Usage**
* essB-attB1 *-F	GGGGACAAGTTTGTACAAAAAAGCAGGCTCATCTTAATGGTGATTTTAACTATG	Cloning of the * essB * deletion mutant in pKOR1 for allelic replacement
* essB *-15codons-R	AAAGATCTTAACATATCTTGCATTTCATTTTTAG	Same as above
* essB *-15codons-F	AAAGATCTCAAAAAGATAAAGAAAAACGCCAAG	Same as above
* essB-attB2 *-R	GGGGACCACTTTGTACAAGAAAGCTGGGTCAGCAACCGTTGGATAATGGTAATTC	Same as above
* essB-Xho *I-F	AAACTCGAGATGGTTAAAAATCATAACCCTAAAAATGAA	Gene expression in * E. coli * or * S. aureus * using pET15b or pWWW412, respectively
* essB-BamH *I-R	AAAGGATCCCTATTTTTTTCTTTCAGCTTCTTGGCGTTT	Same as above
* essB(1–223)-BamH *I-R	AAAGGATCCCTACCCTACTTTGCGTACATATGCATAA	Same as above
* essB(253–444)-Nde *I-F	AAACATATGAAGCATAATGAGCGCATTGAAAAAG	Same as above
* essB(1–252)- BamH *I-R	AAAGGATCCCTATACTGAAAAATATAAAAAGGCTAAAAAT	Same as above
* essB(220–444)-Nde *I-F	AAACATATGCGCAAAGTAGGGCATACCGTTTTCAAA	Same as above
* essB *(Δ224-252)-* EcoR *I-F	AAAGAATTCATGAAGCATAATGAGCGCATTGAAAAAG	Same as above
* essB *(Δ224-252)-* EcoR *I-R	AAAGAATTCTCATGGGTTCACCCTATCAAGCCCTTGCTT	Same as above
* essB-BamH *I-F	AAAGGATCCATGGTTAAAAATCATAACCCTAAAAAT	Production of GST hybrids using pGEX-2TK
* essB-EcoR *I-R	AAAGAATTCCTATTTTTTTCTTTCAGCTTCTTGGCGT	Same as above
* essB(1–223)-EcoR *I-R	AAAGAATTCCTACCCTACTTTGCGTACATATGCATAA	Same as above
* essB(253–444)-BamH *I-F	AAAGGAATCATGAAGCATAATGAGCGCATTGAAAAAG	Same as above

**Table 2:** Strains and plasmids used in this study

**Strains**	**Description**	**Reference**
RN4220	* S. aureus sau1 hsdR * laboratory strain used for passaging plasmid DNA	[34,35]
USA300	Community-acquired methicillin resistant * S. aureus *	NARSA repository [36]
* essB *	USA300 carrying an internal deletion of * essB *	This study
DH5α	* E. coli * K12 * fhuA2 * Δ* (argF-lacZ)U169 phoA glnV44 Φ80 * Δ* (lacZ)M15 gyrA96 recA1 relA1 endA1 thi-1 hsdR17 * for cloning	Our collection
BL21(DE3)	* E. coli * B F^-^ dcm * ompT hsdS *(r_B_^-^m_B_^-^) * gal * for recombinant protein production	Our collection
**Plasmids**	**Description**	**Reference**
pWWW412	pOS1 derivative carrying the constitutive * hprK * promoter, Cm^R^	[33]
pKOR1	temperature sensitive plasmid for allelic replacement, Cm^R^	[32]
pET15b	Vector carrying an N-terminal hexa-histidine repeat followed by a thrombin cleavage site and T7 transcription start; Amp^R^	Novagen
pGEX-2TK	Vector carrying GST followed by thrombin cleavage and phosphorylation sites, and a * taq * promoter; Amp^R^	GE Healthcare
p* essB *	pWWW412 expressing wild-type * essB * codons 1-444	This study
p* essB *^* N *^	pWWW412 expressing codons 1–223 of * essB *	This study
p* essB *^* NM *^	pWWW412 expressing codons 1–252 of * essB *	This study
p* essB *^* C *^	pWWW412 expressing codons 253–444 of * essB *	This study
p* essB *^* CM *^	pWWW412 expressing codons 220–444 of * essB *	This study
p* essB *^* ΔM *^	pWWW412 expressing * essB * lacking codons 224-252	This study
p* his *-* essB *	pET15b expressing histidine tagged * essB * codons 1-444	This study
p* his *-* essB *^* NM *^	pET15b expressing histidine tagged * essB * codons 1- 252	This study
p* his *-* essB *^* CM *^	pET15b expressing histidine tagged * essB * codons 220- 444	This study
p* his *-* essB *^* ΔM *^	pET15b expressing histidine tagged * essB * lacking codons 224-252	This study
p* gst *-* essB *^* N *^	pGEX-2TK expressing GST fused to codons 1–223 of * essB *	This study
p* gst *-* essB *^* C *^	pGEX-2TK expressing GST fused to codons 253–444 of * essB *	This study

### Culture and bacterial fractionation, and western blot experiments

Bacterial strains were grown overnight from isolated colonies in TS broth supplemented with 0.2% serum at 37°C with shaking. Cultures were diluted 1:100 in fresh broth and allowed to shake at 37°C until they reached an absorbance of 1 at 600 nm (A_600nm_) corresponding to exponentially growing bacteria. For whole culture lysates (samples labeled T, for total culture extracts as shown in Figures 2A and 3), cultures (6 ml) were incubated in the presence of lysostaphin (100 μg/ml) for 30 min at 37°C. To separate proteins in the culture medium (M) from those in the bacterial cell (C), cultures (6 ml) were centrifuged (10,000 × * g * for 10 min) and the supernatant was transferred to a new tube prior to lysostaphin treatment of intact cells. For subcellular localization of EssB (Figures 1A and 5 top panel), cultures were centrifuged to separate medium and cells. Staphylococci were washed, and peptidoglycan digested with lysostaphin. Staphylococcal extracts were subjected to ultracentrifugation at 100,000 × * g * for 40 min at 4°C. The supernatant, containing soluble proteins (S), was transferred to a new tube. The sediment containing insoluble membrane proteins (I), was suspended in 6 ml PBS buffer. Proteins in all samples were precipitated with 10% trichloroacetic acid on ice for 30 min. Precipitates were sedimented by centrifugation at 15,000 × * g *, washed, dried and solubilized in 100 μl of 0.5 M Tris–HCl (pH 8.0)/4% SDS and heated at 90°C for 10 min. Proteins were separated on SDS/PAGE and transferred to poly(vinylidene difluoride) membrane for immunoblot analysis with appropriate polyclonal antibodies. Immunoreactive signals were revealed by using a secondary antibody coupled to IRDye^©^ 680. Quantification of western blots was conducted using a Li-Cor Biosciences Odyssey imager. Briefly, cells were grown to the same optical density. All strains reached similar density in the same time period suggesting that either deletion or * cis *-expression of genes did not affect growth of bacteria. Signal intensity of immune reactive signals for EsxA, EssB, EsaB and EsaD was compared to that obtained for WT, WT/vector, * essB */p* essB * or WT/p* essB * sample extracts for Figures 2, 3, 5 A, B, C and D, respectively. Immune reactive signals (as shown in Figure 3) were averaged in three independent experiments and the data was analyzed in pairwise comparisons between WT/vector and variant strains with the unpaired two-tailed Student’s * t *-test and found to be statistically significant.

### Protein and polyclonal antibody purification

Briefly, recombinant EssB, EssB^NM^, EssB^MC^, EssB^ΔM^, tagged with N-terminal hexa-histidine were purified using Ni-NTA Agarose (Qiagen) following manufacturer’s recommendations. Recombinant EssB^N^ and EssB^C^ fused to glutathione S-transferase were purified by using sepharose-immobilized glutathione (Glutathione Sepharose^TM^ 4B, GE Healthcare) following manufacturer’s recommendations. Bound proteins were incubated in 50 mM Tris–HCl buffer (pH 7.5) containing 300 mM NaCl (buffer A) with thrombin (10 U/mg, GE Healthcare) at 4°C for 12 h to cleave the hexa-histidine and gluthathione S-transferase moieties, respectively. Released proteins were dialyzed in buffer B (50 mM Tris–HCl [pH 8.0] containing 150 mM NaCl and 1 mM DTT) and stored at 4°C for use within the next 48 hours. A 100-μl volume of each recombinant protein (~100 μg) was loaded onto a Superdex^TM^ 75 10/300 GL (GE Healthcare) in buffer B at 4°C. The chromatography was performed at a flow rate of 0.5 ml/min, and fractions of 0.5 ml were collected and analyzed by SDS-PAGE. The gel filtration column was calibrated by running a set of protein standards (Aldolase, 158 kDa; Conalbumin, 75 kDa; Ovalbumin, 43 kDa and Myoglobin, 17 kDa). Rabbit polyclonal antibodies raised against full-length EssB were purified prior to use in immunoblot experiments as described earlier [20].

### Transmission electron microscopy (TEM) and image processing

Purified recombinant proteins EssB and EssB^ΔM^ were prepared as described above, dialyzed in Buffer B (without DTT) and diluted to approximately 10 to 50 μg/ml. Proteins were bound to glow discharged, carbon coated (Edwards Auto 306 Evaporator) copper grids (400 mesh), washed, and subsequently negatively stained using 2% uranyl acetate (Electron Microscopy Services). Images were recorded using a Tecnai F30 (Philips/FEI) transmission electron microscope (Field emission gun, 300-kV accelerating voltage, with a magnification of 49,000 to 75,000×) and a high performance CCD camera with a 4k × 4k resolution. Images were captured using Gatan DigitalMicrograph software and processed using Adobe Photoshop (Adobe, San Jose, CA, USA). Images of single protein were selected manually.

## Abbreviations

Ess: ESAT-6 secretion system; T7SS: Type 7 secretion system; Sec: Secretion; LB: Luria Bertani; TS: Tryptic Soy; SDS-PAGE: Sodium dodecyl sulphate polyacrylamide gel electrophoresis; TEM: Transmission electron microscopy microscopy; TM: Transmembrane domain; T: Total culture; M: Medium; C: Cell; S: Soluble proteins; I: Insoluble membrane proteins.

## Competing interests

The authors declare no competing interest.

## Authors’ contributions

YHC conducted most of the experiments in the study and wrote a preliminary draft. MA generated some of the * S. aureus * reagents. APAH performed the transmission electron micrography. DM defined the concept of the study and wrote the manuscript. All authors have read and approved the final manuscript.
